# Electronic Cigarette or Vaping Product Use-Associated Lung Injury in a Previously Healthy Young Male

**DOI:** 10.7759/cureus.18269

**Published:** 2021-09-25

**Authors:** Abdel-Latif S Ismail, Tahir Imaduddeen, Wanis H Ibrahim

**Affiliations:** 1 Internal Medicine Department, Hamad Medical Corporation, Doha, QAT

**Keywords:** tetrahydrocannabinol (thc), nicotine, lipoid pneumonia, acute respiratory failure, eosinophilic pneumonitis, pneumonia, vitamin e, electronic cigarettes, adult respiratory distress syndrome, vaping

## Abstract

Vaping (i.e., the use of electronic cigarettes) has been gaining popularity among people for the past few years, perhaps due to the misconception that its use is less harmful than traditional cigarettes. Although the long-term effects of these products are still unknown, it has been shown that they can be implicated in acute lung injury in healthy people. In 2019, an epidemic of severe acute lung injury was reported in the United States, and it was linked to vaping or electronic cigarette use and was referred to as e-cigarette or vaping product use-associated lung injury (EVALI). Here, we present the first case of EVALI in the state of Qatar.

## Introduction

Vaping refers to the use of an electronic device to administer inhaled drugs, most commonly nicotine and cannabinoids [1].

In 2019, the US Centers for Disease Control (CDC) reported an epidemic of lung disease caused by the use of vape; it was referred to as electronic cigarette (e-cigarette) or vaping product use-associated lung injury (EVALI). There are many patterns of EVALI, and all of them have been linked to recent vaping [2].

E-cigarettes were introduced into Europe in 2006 and the United States in 2007, and since then, the number of their users has been increasing significantly, with rise from 7 million in 2011 to 41 million in 2018 [3]. While this increase has occurred in almost all age groups, the most significant rise has been in adolescents. In 2017, e-cigarettes were the most commonly consumed tobacco products among adolescents [4]. Between 2017 and 2018, there was an increase of 48% and 78% in middle school and high school students who were using e-cigarettes, respectively [5]. This one-year increase is the biggest increase of any substance use tracked by the Monitoring the Future program over the last 44 years [4].

Due to the fact that e-cigarettes are relatively new in the market, there are not many studies conducted on their long-term health effects or safety profile; however, there is a strong correlation between vape use and severe pulmonary injury, which, in some instances, led to admission to intensive care unit and mechanical ventilation [6].

This report describes the first case of EVALI in Qatar.

## Case presentation

A 20-year-old male, with a remote history of childhood asthma with no exacerbations since childhood, presented to the emergency department (ED) with complaints of dry cough and severe shortness of breath that progressed over four days. He did not have fever, chills, chest pain, sputum production, or hemoptysis. There was no preceding upper respiratory infection, gastrointestinal symptoms, or lower limb swelling or pain. He denied having similar symptoms in the past. He gave a history of consuming e-cigarettes for a few months with a recent change in the consumed product and an increase in the amount consumed. He denied having pets or birds at home.

On presentation to the ED, recorded vital signs were temperature: 37.9°C, blood pressure: 99/56 mm Hg, heart rate: 171 beat per minute, respiratory rate: 35 breath per minute, and oxygen saturation: 88% (breathing ambient air). Examination was significant for rapid regular pulse, tachypnea, respiratory distress with the use of accessory muscles of respiration, and bilateral basal coarse crackles without wheezes on chest examination.

Laboratory tests showed leukocytosis, white blood cell count (WBCs) of 30,000/μL with 90% neutrophils, C-reactive protein: 259 mg/L, international normalized ratio (INR): 1.5, serum creatinine: 121 mg/dL, N-terminal pro-B-type natriuretic peptide (NTproBNP): 300 pg/mL, troponin T: 16 ng/L, respiratory viral panel including influenza polymerase chain reaction (PCR): negative, Mycoplasma and Legionella testing: negative, and blood cultures: pending.

Electrocardiogram showed sinus tachycardia, and echocardiography was unremarkable.

**Figure 1:**
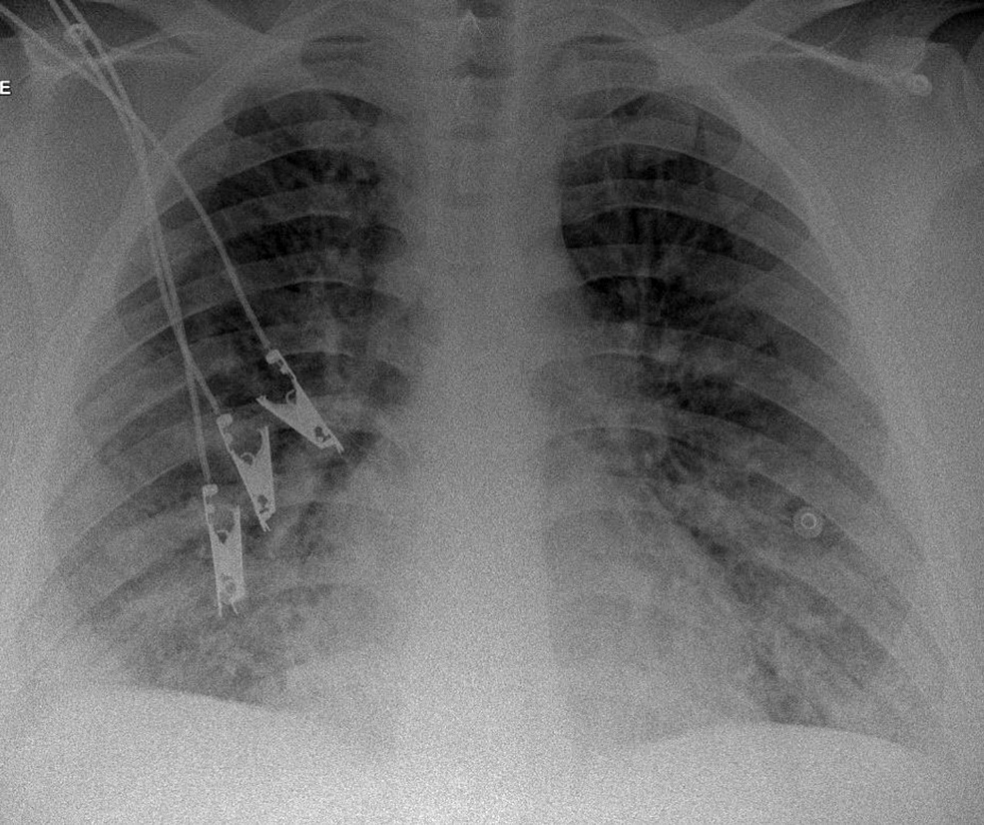
Chest X-ray Bilateral fluffy alveolar infiltrates predominantly affecting lower zones with blunting of both costophrenic angles.

**Figure 2:**
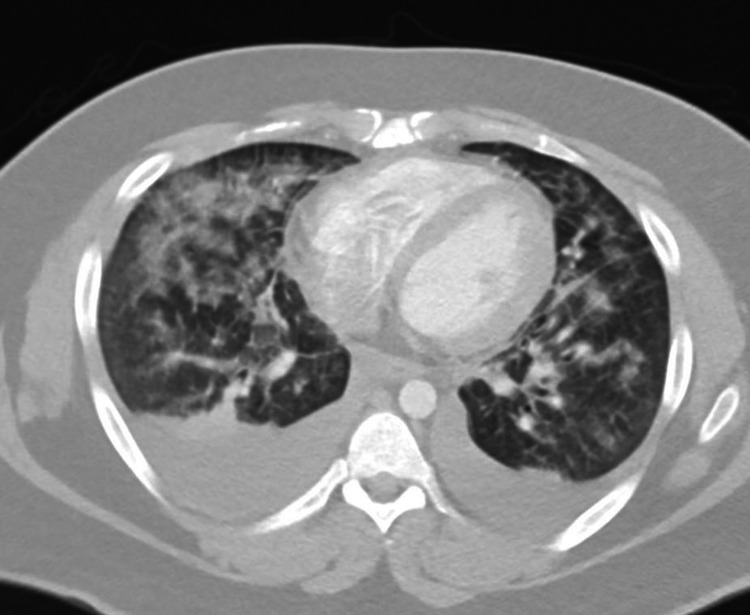
Chest computed tomography (CT) scan with contrast Scattered areas of patchy alveolar airspace opacities, as well as ground-glass opacities and bilateral pleural effusion.

**Figure 3:**
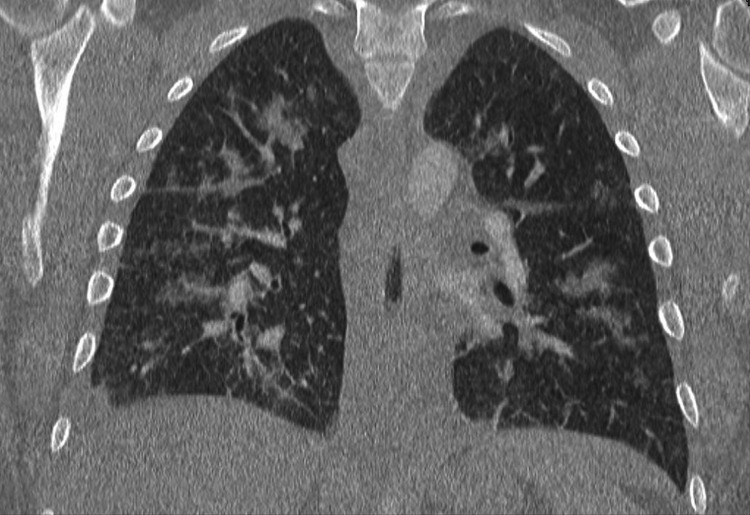
Chest computed tomography (CT) scan with contrast Bilateral perihilar and peribronchial cuffing, with enlarged mediastinal lymph nodes.

Figures 1-3 show the findings of chest X-ray and computed tomography (CT) scan with contrast.

Initially, the patient was treated with oxygen via noninvasive ventilation (NIV), but later, he required endotracheal intubation due to severe tachypnea and hypoxemia and was admitted to the intensive care unit.

His hypoxemia worsened overnight, and repeat chest X-ray showed worsening of the bilateral infiltrates and extension to involve all four quadrants. His oxygen requirement increased, with worsening ratio of arterial oxygen partial pressure (in mmHg) to fractional inspired oxygen (PaO_2_/FiO_2_ ratio) (down to 110 mm Hg), and he was labeled as a moderate acute respiratory distress syndrome (ARDS) case and required proning and extracorporeal membrane oxygenation (ECMO) team consultation.

On the next day, bronchoscopy was done and bronchoalveolar lavage showed pleocytosis with predominant eosinophilia (WBCs: 2,850/µL, eosinophils: 45%), with negative bacterial and fungal cultures and negative viral PCR.

At this point, the working diagnosis was acute eosinophilic pneumonia; thus, treatment with systemic steroids (methylprednisolone 40 mg every 12 hours) was started. His condition dramatically improved within two days, and he was extubated on day 5 of admission and tolerated noninvasive ventilation (NIV) well.

He was subsequently discharged on tapered oral steroids for two weeks. On follow-up in the pulmonary clinic two months later, the patient’s symptoms had resolved completely, and he had quit smoking. Testing for asthma with pulmonary function tests (PFTs) pre- and postbroncholdilator ruled it out, and he was discharged from the clinic. 

## Discussion

The above case discussed the presentation of a young male to the hospital with acute respiratory distress; workup revealed possible e-cigarette or vaping product use-associated lung injury (EVALI), representing the first case of EVALI to be reported in the state of Qatar, and one of the few cases to be reported outside the United States.

The points that were consistent with EVALI diagnosis are as follows:

1. The history of vaping (with recent change in the type and amount of the smoked product).

2. The findings of bilateral fluffy alveolar infiltrates on chest X-ray and ground-glass opacities on chest CT scan.

3. The findings of significant eosinophil count in bronchoalveolar lavage sample.

4. The dramatic improvement in symptoms after administration of systemic steroids.

These are the same points that were used by the CDC in Illinois and Wisconsin in the United States for surveillance of probable/confirmed cases of EVALI in the 2019 epidemic [6].

According to the case series of patients published in Illinois and Wisconsin [6], patients with EVALI most commonly present with cough, dyspnea, fever, chest pain, and diarrhea, and diagnosis is usually suggested by abnormal findings on chest imaging [6,7].

Imaging findings that have been described in EVALI include organizing pneumonia, lipoid pneumonia, hypersensitivity pneumonitis, acute eosinophilic pneumonia, and diffuse alveolar hemorrhage [8,9]. The case presented here can be further subclassified into acute eosinophilic pneumonia EVALI due to the presence of febrile illness for less than four weeks, hypoxemic respiratory failure, diffuse pulmonary infiltrates on chest radiograph, and bronchoalveolar lavage (BAL) with cell count demonstrating more than 25% eosinophils, in addition to the absence of other known causes of pulmonary eosinophilia [10].

Although the cause(s) of the reported lung injuries in the context of vaping remain(s) under investigation, toxic products in e-cigarettes that can potentially injure lung tissues include propylene glycol, glycerin, and nicotine. Identified contaminants include polycyclic aromatic hydrocarbons, nitrosamines, and inorganic chemicals such as toxic metals and flavoring compounds such as diacetyl and 2,3-pentanedione [11-14].

Products that contain tetrahydrocannabinol (THC) are the most commonly reported products among patients diagnosed with EVALI [15]. Additional studies have shown an association between informally acquired prepackaged THC-containing e-cigarettes and EVALI [16]. Analysis conducted by the Food and Drug Administration (FDA) has identified vitamin E acetate in THC-containing e-cigarette products, and testing by the CDC on bronchoalveolar lavage samples from patients with EVALI has identified vitamin E acetate and has not identified vitamin E acetate in control samples [17,18]. These findings suggest an association between vitamin E acetate and lung injury observed in EVALI.

The optimal management of EVALI is still not known. In the large series of 98 patients with EVALI published in Illinois and Wisconsin [5], most patients required oxygen and glucocorticoid therapy, almost one-quarter needed noninvasive ventilation NIV, and another one-quarter needed mechanical ventilation, whereas extracorporeal membrane oxygenation (ECMO) was rarely needed [6,19]. However, we still need controlled prospective studies investigating the efficacy of systemic glucocorticoids to establish their role in the management of EVALI.

## Conclusions

While the prevalence of nicotine consumption has been decreasing around the world for the past two decades, the advent of e-cigarettes and the dramatic increase in the number of their users can indicate a change in the trend toward an increase in overall nicotine consumption.

While e-cigarettes are sometimes thought of as means of quitting smoking, they have not proven their efficacy in that aspect. Furthermore, the presence of additional toxic materials that are not present in traditional cigarettes poses additional health risks.

The case that we have described presented to our hospital a few months after a surge of cases of EVALI in the United States in 2019, and it is among the few cases of EVALI that have been reported outside the United States, and the first case to be reported in the state of Qatar.

This case suggests that it is appropriate to consider EVALI diagnosis in patients presenting with sudden-onset respiratory distress provided that history of e-cigarette smoking is present, especially if vitamin E acetate is a possible ingredient in the vaping product. And thus, chest CT scan and bronchoscopy are among the next appropriate steps in management.

It also highlights the need for obtaining a thorough history in patients presenting with acute respiratory distress, including thorough social and smoking history. It also adds vaping-associated lung injury to the differential diagnosis of acute respiratory distress syndrome.

Furthermore, it reinforces CDC recommendations that doctors should discourage the use of all nicotine products including e-cigarettes, especially those containing THC. Also, individuals should be cautioned to avoid obtaining their e-cigarettes from friends, family, or the illicit market, where product contents can be modified and manipulated easily. Furthermore, vitamin E acetate should not be added to e-cigarettes, as initial studies have linked it to the development of EVALI in patients using e-cigarettes. 
